# Supplementary material for: GeVn complexes for silicon-based room-temperature single-atom nanoelectronics
Source: Sci Rep. 2018 Dec 21;8:18054. doi: 10.1038/s41598-018-36441-w (PMC6303345; doi:10.1038/s41598-018-36441-w)
Supplement: Supplementary file 1 — GeVn complexes for silicon-based room-temperature single-atom nanoelectronics [file 41598_2018_36441_MOESM1_ESM.pdf]

Supporting Informations:

GeV_n complexes for silicon-based room-temperature single-atom nanoelectronics

S. Achilli, N. Manini, G.Onida, T. Shinada, T. Tanii, E. Prati

Thermodynamic versus adiabatic charge transition levels

Figure S.1 displays the structural relaxation around the Ge*V* defect in the D^0 (a), D^- (b) and D^{2-} (c) charge state. This corresponds to the thermodynamic charge transition levels, as reported in the main manuscript.

The arrows show the directions of major displacements of the nearest neighbours of the vacancy with respect to the ideal position. The Cartesian components and modulus of such arrows are reported in Table 1.

A symmetric relaxation toward the vacancy is found in the high spin state D^- (S_{TOT}=1/2) while for the singlet spin state D^{2-} (S_{TOT}=0) the atomic displacement is asymmetric. This behavior is observed also for the neutral defect (S_{TOT}=0) and it is in agreement with expected Jahn-Teller distortion for low spin states.

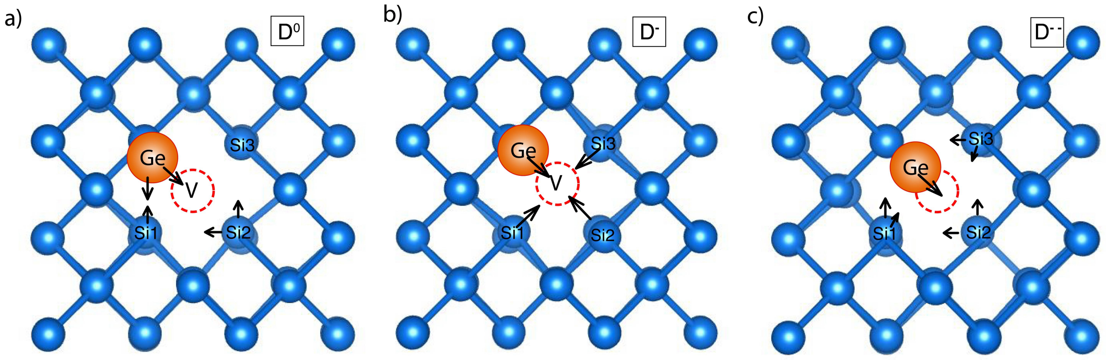

Figure S.1: Atomic relaxation of the GeV defect in the neutral D^0 (a), D^- (b) and D^{--} (c) case.

Moreover, the Ge relaxation toward the vacancy is huge in D^{2-} while in D^- is almost equal to the neutral case. This result, compared to that displayed in Figure 4 of the main manuscript, which shows an increase of the charge density on Ge in the state D^{2-}, is explained in terms of the electrostatic repulsion effect due to the additional electron.

D^0	x (pm)	y (pm)	z (pm)	|**r**| (pm)
Si1	1	17	-11	20
Si2	-10	17	1	19
Si3	-2	-7	-2	7
Ge	18	-34	18	43

D^-	x (pm)	y (pm)	z (pm)	|**r**| (pm)
Si1	15	15	-15	26
Si2	-15	15	15	26
Si3	-15	-15	-15	26
Ge	16	-16	16	28

D^{2-}	x (pm)	y (pm)	z (pm)	|**r**| (pm)
Si1	-3	20	-20	28
Si2	-20	20	-3	28
Si3	-20	3	-20	28
Ge	68	-68	68	118

Table 1: Displacements of the atoms surrounding the vacancy in the three Cartesian directions and total displacement relative to the ideal position.

Figure S.2 reports the charge transition levels for the adiabatic case. The energy calculated for the three GeV_n defects in the adiabatic regime results higher than for the thermodynamic case (See Figure 3 of the main manuscript).

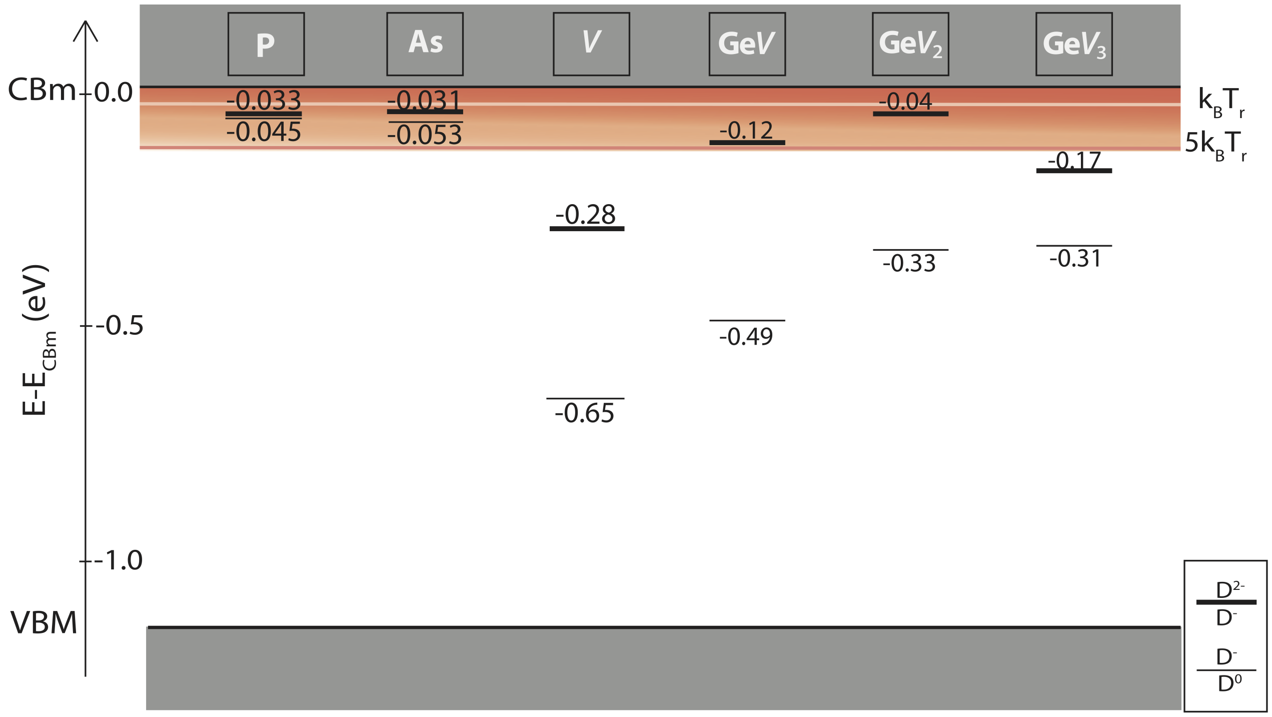

Figure S.2: Adiabatic, i.e. unrelaxed, charge transition levels of the GeV_n clusters. As in Figure 3 of the paper, we report the well-established excited states of conventional dopants and of the isolated single vacancy for comparison.

GeV_n stability

The tendency of Ge to aggregate to vacancy clusters is confirmed by the defect binding energy that we calculate as:

$E_b = E(\mathrm{Ge}V_n) - E(\mathrm{GeSi}_{N-1}) - nE(\mathrm{Si})_{N-1} + NE(\mathrm{Si})$

where $E(\mathrm{GeSi}_{N-1})$ and $nE(\mathrm{Si}_{N-1})$ are the energy of a substitutional Ge and n isolated vacancies in silicon, respectively. N is the total number of silicon atoms in the unit cells.

The values of binding energy, reported in Table 2, are in agreement with previous calculations [1].

	GeV	GeV_2	GeV_3
E_B (eV)	-0.24	-2.5	-4.74
E_F (eV)	3.95	5.95	7.93

Table 2: Binding energy and formation energy of the three defects considered in the D^0 charge state..

The second row of Table 2 reports the formation energy of the three defects.
These increasingly positive energy costs are provided as "damage" energy carried by the ion-implantation process. As a result, in the region where Ge is implanted, one finds a vacancy concentration much larger than the equilibrium concentration compatible with thermodynamics. The subsequent aggregation of vacancies and Ge impurities is driven not only by the energetics reported in the first row of Table 2, but also, and crucially, by kinetics. As a result, GeV defects can easily turn out more abundant than the energetically favored GeV_n defects (n>1).

[1] Chroneos, A.; Grimes, R. W.; Bracht, H. *J. Appl. Phys* **2009**, *105*, 016102
